# Morgan County Medical Society

**Published:** 1867-08

**Authors:** C. T. Wilbur

**Affiliations:** Secretary


### PROCEEDINGS OF MORGAN COUNTY MEDICAL SOCIETY.

The regular meeting of the Morgan County Medical Society was held in Firemen's Hall, on Thursday, June 13th, and was called to order by the President, Dr. Henry Jones, at 2 o'clock P.M.

In the absence of the Secretary, Dr. LUCAS was elected Secretary, *pro tem.*

The minutes of the last meeting, as printed in the Weekly Jacksonville *Journal*, were approved.

The Committee on Examinations reported in favor of Dr. J. G. Cox, of Jacksonville, and Dr. T. N. Stewart, of Exeter, who were at once elected, by the unanimous vote of all the members present.

Dr. Askew, in place of an Essay expected of him, related the history of two cases of phthisis, and their successful treatment with cod liver oil. The patients are both living, and are in apparent good health.

Dr. Prince confirmed the opinion of the beneficial results of cod liver oil in phthisis, considering it a curative as well as a preventive agent, and gave it the preference over the alcoholic treatment.

Dr. Prince had been called in consultation in one of the cases mentioned, and was requested by the Presideut to state some of the signs and symptoms which had resulted in the diagnosis,

Quite a discussion arose as to whether there was pain during the early stages of tuberculosis in the parenchyma of the lungs, and whether pain could arise from inflammation of that tissue.

The President stated that he never had found pain in this disease in his practice, and that the lungs were too insensible an organ to produce it.

Dr. Edgar considered the diagnosis of phthisis in the earlier stage very uncertain, and mentioned as a valuable means of diagnosis the comparative elevation of the scapula on a deep inhalation by the patient; that in the first stages of phthisis, the deposit of tubercular matter at the apex of the lungs, produced similar physical signs, as chronic bronchitis, extending by continuity to the adjoining parenchyma, rendering the lung imperveous and flat, or dull on percussion; that the principle diagnostic symptom was the existence or absence of pain attending the primary deposit of tubercles, whereas pain did accompany the other condition to a greater extent.

Dr. Buckner next read an essay on the Practice of Medicine, reviewing the comparative progress of this with other sciences.

On motion, the thanks of the Society were tendered Dr. Buckner for this interesting paper.

Dr. Edgar then read an Essay on "Veratrum Viride," giving some valuable accounts of its history, and therapeutic properties as an arterial sedative, the doses, its different action, when used in the mouth, and as a subcutaneous injection. The reason that it is not much more used, is, that Dr. Norwood, of South Carolina, who brought it in general use, recommended it unwisely as an emetic, and in some diseases in which it is not applicable. The U. S. Dispensatory, the most popular standard work, recommends its use in over-doses, which often produces harm and anxiety both to patient and physician.

Dr. Edgar was requested by the Society to furnish his interesting paper to some medical journal for publication.

The therapeutic properties of veratrum viride gave rise to the description of a new instrument, the sphygmograph, or pulse-writer, and its value as a diagnostic agent in the action of medicine on the pulse; for instance, the effect of the administration of alcohol as an antidote to veratrum viride. According to Dr. Edgar, the tincture of cantharides, in connection with alcoholic stimulants, is the best restorative in case of an over-dose. The peculiarities of the action of alcohol are, that small doses increase the pulse in frequency, and large doses increase it in volume, force, and fullness, and not in frequency; also, that it reduces the temperature of the body, as shown by numerous experiments under the use of the thermometer, applied in the axilla or under the tongue.

Dr. Craig asked a question in regard to the antagonistic effect of belladonna in opium poisoning, and *vice versa.* He and Dr. Edgar gave cases, in which they derived apparent good results, yet the treatment was combined with other remedies.

Dr. Craig reported, also, a case of dysentery treated successfully by veratrum viride. The administration of the agent was accidental.

Dr. Reed spoke of the therapeutic effects of several drugs similar in action to veratrum viride as arterial sedatives.

On motion to appoint a committee to revise the Constitution of the Society, the President appointed Drs. Wilbur, Craig, Prince, Bibb, and Askew.

The President appointed Drs. Bibb and Craig to read Essays at the next meeting.

On motion, the Society adjourned to meet on the second Thursday in July, at two o'clock precisely, at Firemen's Hall, in Jacksonville. C. J. LUCAS, M.D., Secretary, *pro tem.*

—

The Society met in Firemen's Hall, in Jacksonville, on Thursday, the 11th of July, at 2 P.M.

Dr. Henry Jones, the President, called the meeting to order, when the minutes of the June meeting, as printed in the Weekly Jacksonville *Journal,* were read and accepted.

Dr. William S. Edgar, one of the committee of five on legislation, appointed by the State Medical Society, suggested the consideration, by the Society, of a proposed law to regulate, in the State of Illinois, the practice of medicine, to protect the people of the State more effectually from the ignorance of the many illiterate and uneducated self-styled doctors who are found throughout the country.

On motion, it was made the subject for discussion at the next regular meeting.

Dr. Bibb reported a case of gonorrhœal orchitis, treated by a deep incision, followed by immediate relief to the patient, and a more speedy convalescene than by any other method of treatment that he had ever adopted. The Doctor had been prompted to try this method from the successful results of similar operations by eminent men in Europe and in this country, who had published their plan of treatment in late medical journals, and was much pleased with the result of the practice in the case referred to.

Dr. Prince remarked, though he had never tried the plan, yet it was recommended by many surgeons, and was uniformly well spoken of. He supposed the practice was based upon the hypothesis that the fluids discharged by the puncture had given rise to the pain by pressure, and the removal of the pressure gave immediate relief.

Dr. Kimber, of Waverly, mentioned a case where he had accidentally punctured this organ, but that immediate relief to the patient was the result, and a speedy convalescence. The patient had been troubled with orchitis and chronic hydrocele for several years, but after the accidental operation recovered completely, and had never been troubled since.

Dr. Edgar had found but little difficulty in treating this disease by the ordinary method.

Dr. Wilbur had been successful in several cases by using tartar emetic in nauseating doses, producing speedy convalescence.

Dr. Craig, of Arcadia, then read a paper on scarlet fever, giving the history of a recent epidemic of this disease in his sphere of practice.

The Doctor was requested by the Society to publish his Essay in some medical journal.

Dr. Craig had found that nitrate of silver in solution, applied by spray through the atomizing tube; also, a strong infusion of capsicum, applied in the same way, very beneficial local applications to the tonsils and pharynx. Used, also, tincture of muriate of iron with glycerine, as a local application to the throat.

Dr. Prince suggested that the way in which this epidemic had originated was additional evidence of the contagiousness of the disease, and should be remembered.

Dr. Henry Jones, in relation to the imaginary prophylactic power of belladonna in this disease, remarked that in one family where he had faithfully tested its use, one little unruly boy had resisted every effort of his parents to make him take the belladonna, and he alone escaped the disease. He had found dilute muriatic acid locally applied the best remedy, and considered chloroform and chlorate of potash valuable remedies in connection with other means of treatment. He had observed that there was greater acceleration of the pulse in scarlet fever than in any other of the acute febrile diseases.

The Examining Committee reported favorably in the case of Dr. Skinner, of Bethel, when he was unanimously elected a member of the Society.

Dr. Brown, of Waverly, Dr. Dutton, of Jacksonville, Dr. DeLeuw, of Jacksonville, and Dr. C. A. Edgar, of Arcadia, were appointed to prepare Essays for the next meeting.

After the usual remarks of mutual admiration and congratulation, the Society, at five o'clock P.M., adjourned to meet at two o'clock P.M., on Thursday, the 8th of August.

C. T. Wilbur, M.D., Secretary.

